# A Decision Tree Cost Analysis of Intracranial Bleed Detection Using a Near-Infrared Device Across Various Healthcare Levels

**DOI:** 10.3390/jmahp14020033

**Published:** 2026-06-01

**Authors:** Mamta Patel, Amit Kumar Mittal, Mohit Agrawal, Oshima Sachin, Kavitha Rajsekar, Bharat Choudhary, Suryanarayanan Bhaskar, Kuldeep Singh

**Affiliations:** 1 Resource Centre, Health Technology Assessment, All India Institute of Medical Sciences (AIIMS), Jodhpur 342001, India; mamtaavu@gmail.com (M.P.); amitkrbiotech@gmail.com (A.K.M.); 2 Department of Neurosurgery, All India Institute of Medical Sciences (AIIMS), Jodhpur 342001, India; ma.nsurgeon18@gmail.com (M.A.); bhaskars@aiimsjodhpur.edu.in (S.B.); 3 Department of Health Research, Ministry of Health & Family Welfare, Health Technology Assessment in India (HTAIn), New Delhi 110001, India; oshima.dhr@gmail.com (O.S.); kavitha.rajsekar@nic.in (K.R.); 4 Department of Trauma and Emergency, All India Institute of Medical Sciences (AIIMS), Jodhpur 342001, India; drbharatpaeder@gmail.com; 5 Department of Pediatrics, All India Institute of Medical Sciences (AIIMS), Jodhpur 342001, India

**Keywords:** traumatic brain injury, intracranial haemorrhage, near-infrared spectroscopy, cost analysis, incremental cost

## Abstract

Traumatic brain injury (TBI), mainly caused by road traffic accidents, is a serious global public health concern. Computed tomography (CT) is the best way to detect intracranial haemorrhage (ICH), but it is not always feasible because it is hard to access, exposes people to radiation, and is expensive, especially in low- and middle-income countries. Portable near-infrared spectroscopy (NIRS) devices offer a non-invasive, point-of-care option for early detection of ICH. The objectives of this study were to estimate the cost per case detected for patients with mild-to-moderate TBI, to estimate incremental cost and to perform a budget impact analysis to assess the financial feasibility of implementing this technology. This study employed a decision tree model from a health system perspective to calculate the cost per detected case and the incremental cost of NIRS across three tiers of care: ambulances, community health centres (CHCs), and tertiary hospitals. The cost per mild-to-moderate TBI case found was Rs. 2177.90 in ambulances, Rs. 748.09 in CHCs, and Rs. 628.14 in tertiary hospitals. The extra cost per patient was Rs. 984.15, Rs. 360.90, and Rs. 289.78, respectively. At the system level, NIRS raised the total costs for 264 ambulance patients from Rs. 37.71 lakh to Rs. 40.31 lakh and for 858 CHC patients from Rs. 115.64 lakh to Rs. 118.73 lakh. National extrapolation indicates a first-year budgetary impact of approximately Rs. 442 crores for ambulances and Rs. 187 crores for CHCs. These results support the strategic, phased implementation of NIRS to use resources better and improve early diagnosis of TBI.

## 1. Introduction

One of the rapidly escalating public health problems worldwide is attributed to traumatic brain injury (TBI) due to road traffic accidents [1]. TBI is classified as a form of acquired brain injury that can be diagnosed within the first 24 h of the injury or at the time of the accident. TBI is caused by external mechanical forces, including road traffic accidents, falls, assaults, interpersonal violence and sports injuries. The severity of the injury, symptoms can include headaches, loss of consciousness, confusion, vomiting, seizures and neurological impairments [2]. It has the highest incidence of all common neurological disorders and is now recognised as both an acute and chronic disorder with long-term implications, including a higher risk of late-onset neurodegeneration [1,2]. According to the 2019 Global Burden study, about 90% of the 4 million injury-related deaths worldwide happened in low- and middle-income countries (LMICs), and autopsy results indicate that a significant number of these deaths are attributable to TBI [3]. Over the next several years, it is anticipated that the burden of injuries in LMICs will increase. Some of the research indicates that over a million trauma-related deaths occur in India annually, with 50% of those deaths being connected to TBI [4].

A prompt and precise diagnosis is essential for patients with TBI to receive early treatment. A head CT (Computed Tomography) scan is the preferred examination for identifying intracranial haemorrhage (ICH). However, the concerns for CT scans are limited access, increased radiation exposure, and an inappropriate burden on healthcare resources [5]. Thus, emergency medical services or the trauma department must swiftly triage trauma patients for additional testing and care. Field triage can help identify whether a patient has an intracranial haematoma and, if so, screen them for a CT scan referral. CT scans are frequently overused in mild head injury patients for cautionary purposes, resulting in unnecessary radiation exposure and increased healthcare expenses. Accurate, user-friendly, and quick-to-use portable ICH detection technology can reduce the number of pointless CT scans while also signalling the urgent need for one in TBI patients [6].

Machine learning-enabled near-infrared spectroscopy (NIRS) provides an objective, non-invasive approach for the early detection of intracranial haemorrhage in traumatic brain injury, including both presymptomatic and delayed-bleed cases [7]. The technology is radiation-free, allowing for repeated use in vulnerable populations such as pregnant women and infants, and is designed for rapid interpretation by paramedics and frontline clinicians. Its portable, bedside capability enables real-time monitoring in prehospital settings, emergency departments, and inpatient wards [6,8,9]. There are a few limitations of this NIRS device, such as limited penetration and limited accuracy to fully replace CT.

In light of these challenges and the growing need for rapid, resource-efficient triage tools for traumatic brain injury, the present study was designed to evaluate the impact of a portable NIRS-based intracranial bleed detector. Specifically, we aimed to (i) estimate the cost per case detected for patients with mild-to-moderate TBI, (ii) estimate incremental cost, and (iii) perform a budget impact analysis (BIA) to assess the financial feasibility of implementing this technology at the ambulance and community health centre (CHC) levels of care.

## 2. Materials and Methods

### 2.1. Economic Model

A decision tree model was developed to analyse the cost of portable NIRS instrument to identify ICH in patients with mild-to-moderate TBI [10]. The model is India-specific and has been approved by clinical and subject-matter specialists. It contrasted two diagnostic strategies: (i) standard care without NIRS and (ii) treatment with NIRS-assisted technology, followed by confirmation CT scans if needed.

Three healthcare tiers were included in the decision tree: tertiary hospital, CHC, and ambulance (prehospital). A simulation of the real-world caseloads was used for each level. Clinical pathways based on NIRS diagnostic accuracy (true positives, false negatives, false positives, and true negatives) were represented by the model’s branches. The model depicts a mixed rural–urban healthcare utilisation pattern rather than a solely urban or rural population. Each branch was assigned costs and probabilities so that the incremental cost of each new case found could be estimated.

### 2.2. Intervention and Comparator

The intervention involved an NIRS-based intracranial bleed detector. This technology is non-invasive, portable, radiation-free, and intended for use by healthcare personnel at various levels of care. Its primary function is to detect ICH early on and triage patients for CT referrals. The comparator was standard treatment without NIRS, in which suspected TBI patients were referred for CT scanning based purely on clinical assessment. Regardless of the cause of injury, all mild-to-moderate TBI cases requiring diagnostic examination were included in the model.

### 2.3. Outcomes

The primary outcome is to estimate the incremental cost per case detected by a non-invasive, portable NIRS intracranial bleed detector for patients with mild-to-moderate TBI.

The secondary outcomes were: (i) total care costs for the patient cohort at each level, (ii) cost differentials between intervention and comparator arms, (iii) a national-level BIA to estimate the budgetary consequences of implementing NIRS technology in ambulances and at CHCs. By combining diagnostic accuracy measures (sensitivity and specificity) with cost data, the model provided a comprehensive evaluation of NIRS technology’s clinical utility.

### 2.4. Input Parameters

The study’s parameters were obtained from the published literature through targeted reviews and, where required, systematic searches. PUBMED, EMBASE, Scopus, the Cochrane Central Register of Controlled Trials (CENTRAL), the Cochrane Database of Systematic Reviews, the NHS economic evaluation database for Systematic Reviews, meta-analysis, randomised clinical trials (RCTs), observational studies and the CEA registry for economic evaluations using MESH-specific terms were among the online search engines that were used to conduct the literature review [11].

Epidemiological parameters, including TBI prevalence and the distribution of mild/moderate cases, were obtained from the Global Burden of Disease study and Indian trauma registries. The Niti Aayog report 2021 and Rural health statistics 2021–2022 provided data on the number of ambulances, CHCs, and tertiary hospitals. Referral pathways and clinical management algorithms were developed through structured consultation with domain experts and frontline clinicians. The model input parameters are summarised in Table 1.

### 2.5. Costing Data

The analysis adopted a health-system perspective, accounting for both direct medical and system-level expenses. The following cost categories were incorporated: Capital cost—initial investment necessary to acquire the device; Operating cost—continuing expenses associated with the device’s use; Consumable cost—any disposable material that is required for operating this device; Training cost—training healthcare staff to utilise the technology effectively; and Maintenance costs—include routine maintenance and servicing of the device to ensure its optimal performance. To determine these cost parameters, we consulted experts and used a combination of secondary data sources. This allowed us to see clearly how much it would cost to deploy the technology at various levels of the healthcare system.

Table 2 shows the costs of the model for transportation, service delivery, therapy, diagnostic imaging, and referrals. These estimates were used as economic inputs for the decision tree model. All costs were reported in Indian rupees (INR), inflated to 2024 values using an annual inflation rate of 5.5%, and discounted at 3% to reflect standard health economic practice [19,20].

### 2.6. Model Analysis

The cost analysis was performed in two sequential stages. First, a base-case analysis was conducted to estimate the incremental cost per ICH case detected at each level of care, ambulance services, CHCs, and tertiary hospitals by dividing the difference in total costs between the NIRS strategy and standard care by the additional number of ICH cases identified. Setting-specific patient cohorts were modelled for each healthcare level.

Second, a BIA was undertaken by extrapolating the model to the national level using estimates of the number of ambulances and CHCs in India. This step assessed the first-year financial impact of large-scale adoption of NIRS technology within the public health system.

### 2.7. Assumptions

Referral Pathways with NIR Technology: Patients with a positive NIR test for intracranial bleeding were assumed to be referred directly to a neurosurgical centre for specialised management. At the same time, those with a negative result were initially managed at a district hospital and referred onward only if further clinical evaluation indicated the need for higher-level care.

Cost and Budget Considerations: The study assumes that the number of patients will not change and that the population across all healthcare facilities will remain the same. Budget estimates at the national level are intended to reflect the average system-wide costs. However, they do not take into account changes in infrastructure, regional differences, and NIR devices that can affect the economy in the real world [27].

### 2.8. Ethics Statement

The article is founded on the studies that have already been conducted. No new research involving people or animals has been carried out by any of the authors. All data used in this study were obtained from publicly available sources and published literature.

## 3. Results

### 3.1. Model Input Parameters

Table 1 and Table 2 illustrate the key epidemiological and cost inputs used in the model. All the data parameters are based on prior published literature. We meta-analysed the results of three Indian studies on diagnostic performance using Meta-XL. The pooled sensitivity of the NIRS device was 95% (95% CI: 93–96%), and its specificity was 91% (95% CI: 80–99%) [6,8,9]. Sensitivity and specificity estimates were pooled from published studies that evaluated NIRS devices for cerebral haematoma detection using similar near-infrared spectroscopy technology. These details are displayed in Supplementary Figures S1 and S2, which shows the forest plot. These values were applied consistently at the levels of healthcare to model the diagnostic accuracy.

The costs associated with service delivery at CHCs and tertiary hospitals, mild and moderate TBI treatment, head CT scans, referrals and transportation, NIRS implementation, including training and maintenance of devices, were also included in the model. All costs were adjusted to 2024 INR prices and subsequently discounted using general health economic principles. Supplementary Tables S1–S3 show that the NIRS-based detection strategy costs Rs. 2177.90 per case in ambulances, Rs. 748.09 at CHCs, and Rs. 628.14 at tertiary hospitals.

### 3.2. Cost Analysis at Different Health Care Levels

**Figure 1:**
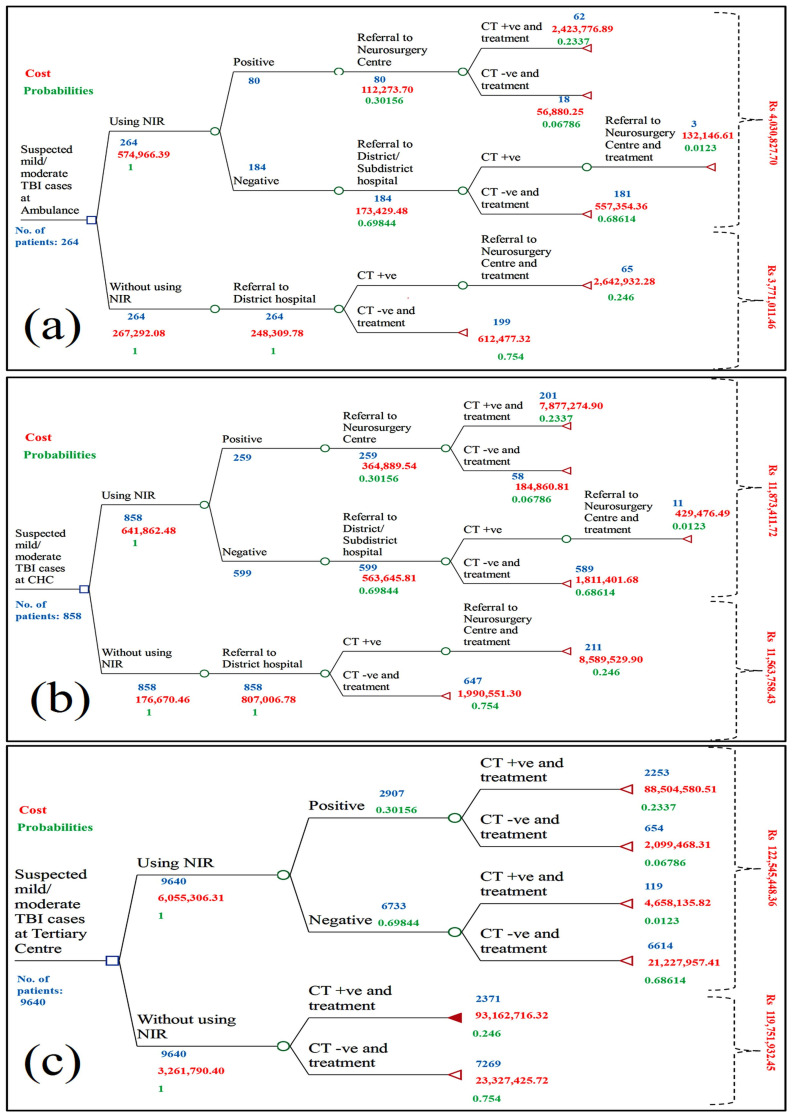
Decision tree model: (**a**) Model for ambulance level; (**b**) model for CHC level; (**c**) model for tertiary level.

Ambulance level: A cost analysis compared the NIRS-based diagnostic strategy with standard care (without NIRS) in the ambulance setting, using a cohort of 264 patients. Two scenarios were modelled: (i) triage with NIRS for mild-to-moderate TBI and (ii) conventional management without NIRS (Figure 1a). The incremental cost per patient was calculated as the difference in total costs between the two strategies divided by the cohort size, resulting in an additional cost of Rs. 984.15 per patient associated with NIRS implementation (Table 3).

CHC level: A cohort of 858 patients was modelled to assess the cost of implementing NIRS for the diagnosis of mild-to-moderate TBI. Two strategies were compared: standard care and NIRS-assisted triage (Figure 1b). The incremental cost per patient was estimated as the difference in total costs between the two strategies divided by the cohort size, resulting in an additional cost of Rs. 360.90 per patient associated with NIRS adoption (Table 3).

Tertiary level: Two strategies were compared at the tertiary care level: standard care and NIRS-assisted diagnosis (Figure 1c). The incremental cost per patient was calculated by dividing the total cost difference between the two strategies by the cohort size of 9640 patients, resulting in an additional cost of Rs. 289.78 per patient associated with NIRS implementation (Table 3).

### 3.3. Comparative Cost Analysis Across Healthcare Levels

Overall, the decision tree analysis demonstrates substantial variation in the cost of the intracranial bleed detector across healthcare settings (Table 3). The decrease in the incremental cost between the ambulances and CHCs and tertiary hospitals indicates that the cost of high levels of care is increasingly becoming affordable. The incremental cost per patient at the tertiary centres was the lowest, and this can probably be attributed to the fact that they already had the necessary tools to diagnose the patients and could save money by attending to more patients simultaneously. These outcomes indicate that more advanced facilities have a higher ability to utilise the advanced diagnostic technologies positively.

### 3.4. Budget Impact Analysis

A BIA was performed to estimate the national-level financial implications of implementing an NIRS at both ambulance and CHC levels, as shown in Supplementary Table S4.

BIA at ambulance level: BIA demonstrated a substantial economic burden associated with adopting NIRS. For a representative cohort of 264 patients requiring intracranial bleed detection, the total cost of standard care was estimated at Rs 3,771,011.46. In comparison, the cost associated with implementing NIRS was estimated at Rs 4,030,827.70, resulting in an incremental cost of Rs 259,816.24. Given the presence of approximately 17,000 ambulances across India (Emergency and Injury Care at Secondary and Tertiary Level Centres in India: A Report of Current Status on Country Level Assessment by NITI Ayog 2021) [15], the projected national budget impact in the first year of implementation at this level would be approximately Rs 442 crores.

BIA at the CHC level: The financial impact differs in scale. For a population of 858 patients, the standard care cost was calculated at Rs 11,563,758.43, while the cost with NIRS integration rose to Rs 11,873,411.72, resulting in an incremental cost of Rs 309,653.29. With 6064 CHCs operational nationwide (Rural health statistics 2021–2022, GOI, Ministry of Health and Family Welfare) [17], the estimated first-year budget impact of introducing the NIRS at CHCs amounts to Rs 187 crores.

While adding NIRS to TBI diagnostics appears likely to yield better results, the initial costs are high at both levels of care. These results show how important it is to plan and prioritise finances carefully when expanding new diagnostic technologies in healthcare systems with limited resources [28].

## 4. Discussion

NIR technology for detecting intracranial bleeding represents a significant advancement for healthcare systems, particularly in resource-limited settings. NIR technology offers several benefits over traditional methods. For example, it is non-invasive, portable, and can be used in prehospital settings. The NIR device’s 95% sensitivity and 91% specificity indicate it could be useful for detecting intracranial haemorrhages. This could help patients by allowing for faster treatment and fewer unnecessary CT scans. This is crucial given the high incidence and potentially severe outcomes associated with TBI, particularly in LMICs, where the burden of such injuries is substantial [2].

The clinical utility of NIR technology for diagnosing TBI has been the subject of numerous prior studies, particularly as a point-of-care screening tool. To detect cerebral haematomas in prehospital settings, Robertson et al. evaluated a handheld NIR spectroscopy equipment in a clinical trial [29]. The study’s high sensitivity (88%) and specificity (90.7%) justify its usage as a triage tool in field or resource-constrained settings. The lack of an economic analysis in that study, however, limited the capacity to evaluate the wider financial effects of NIR adoption.

According to research, NIR technology can speed up diagnosis and prevent needless CT scans in emergency rooms, particularly in situations of mild traumatic brain injury [30]. However, their analysis did not consider its utilisation across various levels of health care or evaluate the broader national cost implications. Our analysis fills this gap and offers a more comprehensive understanding of implementation scalability and affordability by assessing costs from the first point of contact (ambulance) to secondary and tertiary levels. The ambulance transportation costs were derived from Prinja et al. (2013) and are relevant to the Indian healthcare context, as the average cost of transportation [25].

The cost analysis shows that the incremental cost per patient of employing NIR technology varies widely across healthcare settings. The cost of ambulance services is significantly higher than that of community and tertiary health centres. This increase in ambulance expenditures is mostly due to additional costs associated with equipping a large number of ambulances nationwide. Trauma patient triaging or classification is primarily performed in hospital settings. However, there is an increasing demand for field-deployable technologies that can identify cerebral haematomas in prehospital settings, such as ambulances, without requiring expert interpretation. The potential benefits of NIR technology in ambulances, such as enhancing triage accuracy and optimising emergency care, make the investment worthwhile, particularly in situations where immediate intervention is crucial to patient outcomes.

In contrast, our study uses decision tree analysis and BIA to give both diagnostic performance data and extensive cost modelling. Our meta-analysis pooled sensitivity (95%) and specificity (91%), which are slightly greater than those reported in prior research, suggesting that the model examined here may have improved in performance. These improvements in diagnostic accuracy may affect subsequent clinical decisions, such as faster referrals and fewer unnecessary imaging tests, altering the economic dynamics of its use.

At the CHC level, the cost per patient is more manageable than that of ambulances; nonetheless, the total annual budget impact remains significant due to the larger number of CHCs. Tertiary health centres incur the lowest incremental costs per patient. The reduced cost per patient at this level is due to the established infrastructure and economies of scale that tertiary facilities receive. These facilities are designed to handle large patient volumes and integrate new technologies more effectively. Our findings contribute to the existing clinical data on NIRS by providing an economic viewpoint at different levels of the healthcare system. Previous research has mostly focused on diagnostic accuracy, whereas our findings show that the economic consequences of deploying NIRS vary significantly by setting. The reduced incremental costs observed in the tertiary care centre indicate that economies of scale and existing infrastructure play an important role in cost efficiency. In contrast, greater ambulance prices reflect the additional expenditure needed for widespread deployment. These findings emphasise the value of context-specific implementation strategies.

Research on low-resource settings, including Ethiopia, has highlighted that resources can be used to build trauma care systems and enhance prehospital systems, specifically ambulance services and first responder training, to increase survivability rates in cases of traumatic brain injury [31]. Equally, studies conducted in other low-income nations show that access to CT scans is low and often inaccessible when required, leading to delays in diagnosis and proper referral. Such findings bring out the inherent problems that a health system encounters due to inadequate infrastructure and a lack of state-of-the-art imaging [32]. Such results echo the Indian setting, where such gaps exist, especially in rural and semi-urban regions. The addition of portable diagnostic methods, including NIRS, to these environments may be a complement to more comprehensive system-wide interventions by helping to identify TBI cases promptly and refer them to care, as well as meeting the urgent goal of trauma care capacity development.

From a policy standpoint, our findings suggest that a phased implementation plan may be more appropriate, prioritising higher-level facilities with greater cost efficiency before expanding to prehospital settings. This approach could aid in optimising resource allocation in resource-constrained healthcare settings. In contrast to earlier research that focused solely on clinical performance, our analysis incorporates cost modelling and diagnostic accuracy, providing a more comprehensive foundation for decision-making.

Roldan et al. reported a thorough evaluation of NIRS initial use in TBI monitoring [33]. Despite the fact that NIRS is typically applied to measure cerebral oxygenation in patients with severe TBI, particularly in intensive care units, there is an increased interest in its diagnostic capabilities. Based on their review, NIRS can become a non-invasive technology, yet it is currently burdened by methodological and technical issues, including the issue of specificity, device calibration, and consistency. This aligns with our findings, which revealed that the NIRS device we reviewed was good in terms of sensitivity and specificity, yet it was not reliable enough to replace CT imaging to achieve a conclusive diagnosis. This study did not evaluate the accuracy of real-world triage. Diagnostic ambiguity was taken into account by the sensitivity and specificity measures. Confirmatory CT is still needed to minimise the possibility of misclassification and NIRS can be regarded as an additional instrument to aid clinical decision-making.

Due to model-based cost evaluations, this study has several possible sources of uncertainty. These include changes in patient volume across healthcare levels, disparities in device costs and maintenance, estimates of diagnostic accuracy, and presumptions about healthcare utilisation and referral patterns. The overall cost estimates and cost disparities between healthcare settings may be impacted by changes in these characteristics. Such uncertainty should be taken into account when planning large-scale implementation in real-world policy decisions.

There are a few limitations with this technology. NIR technology does not have the sensitivity and specificity to completely replace CT scans. CT is still the best way to make a final diagnosis and plan treatment, even though it is a useful extra tool. Future improvements may make it a better standalone diagnostic test. There is no current data on how NIR technology affects the quality of life, morbidity, and mortality of TBI patients. Some included input cost data were decade old, and although inflation adjustments were applied, they may not fully reflect structural changes in healthcare delivery, technological advancements, or evolving treatment patterns. Prospective studies are necessary to ascertain its effectiveness in enhancing patient outcomes, including mortality rates and recovery trajectories.

## 5. Conclusions

The integration of NIRS for detecting intracranial bleed has various cost implications across different levels of the healthcare system. These results indicate the economic constraints of adopting NIR technology in different healthcare contexts. Decision-makers should weigh these expenses against the possible advantages of improved diagnostic mechanisms, both the short-term cost in the immediate effect and the long-term advantages of early and accurate TBI diagnoses. NIR technology to be implemented in the healthcare system in a financially sustainable manner, proper planning and resource allocation are necessary.

## 6. Recommendations for Policy Implications

NIR technology does not substitute CT, and rather, it is a non-invasive triage technique that complements CT by facilitating the identification and prioritisation of intracranial bleeding before the imaging process. It assists with the quick prehospital screening and directing urgent care in ambulances. In CHCs, it helps to make timely referrals to neurospecialty centres, and in tertiary hospitals, it expedites the initiation of CT, enhances patient traffic, and can decrease unwarranted repeat imaging.

## Figures and Tables

**Table 1:** Model input parameters and data value(s).

Input Parameter	Value	Source (Reference)
Prevalence of TBI in India	0.92%	Adusumilli et al. 2023 [12] and GBD ^1^ data [13]
Percentage of mild/moderate TBI patients in total TBI patients	77.7% (51.42% mild + 26.28% moderate)	Singh et al. 2018 [14]
Prevalence of mild/moderate TBI patients in 1 lakh population	715	Calculated (920 × 77.7%)
No. of ambulances in India	17,000	Niti Aayog report 2021 [15]
Daily emergency ambulance calls in India	50,000	GVK EMRI data from the user department [16]
Probability of TBI cases in the ambulance	25%	GVK EMRI data from the user department [16]
TBI patients seeking services at the ambulance (per year)	264 (22 per month)	Calculated
Number of CHCs in India	6064	Rural health statistics 2021–2022 [17]
Population catered by CHC	120,000	Rural health statistics 2021–2022 [17]
Prevalence of mild/moderate TBI at the CHC level	858	Calculated from the CHC population (715/100,000 × 120,000)
The population catered to by the tertiary hospital	1,348,138	Calculated (district population ÷ no. of tertiary hospitals)
Prevalence of mild/moderate TBI in a tertiary hospital	9640	Calculated (715/100,000 × 1,348,138)
Probability of suspected TBI patients showing positive CT scan results	24.60%	Maharjan et al. 2017 [18]

^1^ GBD: Global Burden of Disease data.

**Table 2:** Model input parameters related to cost used in the analysis.

Input Parameter	Base-Case Value (INR)	Lower Limit (INR)	Upper Limit (INR)	Year 2024 Cost (After Inflation) (INR)	Source (Reference)
Unit cost of delivering services at CHC	185	109	349	205.91	Chauhan et al. 2022 [21] & CHSI database [22]
Unit cost of delivering services at a tertiary care hospital	304	223	433	338.36	Chauhan et al. 2022 [21] & CHSI database [22]
Unit cost of a patient in the treatment of a minor head injury	7800	–	–	–	Manmohan et al. 2006 [23]
Unit cost of a patient in the treatment of moderate head injury	22,172	–	–	–	Manmohan et al. 2006 [23]
Unit cost of a patient for minor/moderate head injury	14,986	–	–	39,285.29	(7800 + 22,172)/2 (calculated from Manmohan et al. 2006) [23]
Unit cost of head CT at a tertiary hospital	2871	–	–	2871.00	CHSI database [22]
Health system cost for referral of a patient to the neurosurgery centre/district hospital	1001	801	1201	–	Beena Nitin Joshi et al. 2021 (lower limit assumed for district hospital, upper limit for neurosurgery centre) [24]
Health system cost for referral of a patient to the neurosurgery centre	1201	–	–	1410.26	Beena Nitin Joshi et al. 2021 [24]
Health system cost for referral of a patient to the district hospital	801	–	–	940.57	Beena Nitin Joshi et al. 2021 [24]
The cost of transport via ambulance	561.83	462.81	673.77	1012.47	Prinja et al. 2013 [25]
The treatment cost of a mild/moderate TBI patient when the CT is negative	3672	–	–	3811.57	Calculated (unit cost of CT + health system cost)
Inflation rate	5.50%	–	–	–	WorldData [26]
Discounting rate	3%	–	–	–	Malaisamy Muniyandi et al. 2022 [20]

**Table 3:** Various input parameters and their cost/value(s).

Setting	Parameter	Cost/Value (INR)
Ambulance	Total cost of intervention	4,030,827.70
	Total cost of the comparator	3,771,011.46
	Number of patients	264
	Incremental cost per patient	984.15
CHC	Total cost of intervention	11,873,411.72
	Total cost of the comparator	11,563,758.43
	Number of patients	858
	Incremental cost per patient	360.90
Tertiary care setting	Total cost of intervention	122,545,448.36
	Total cost of the comparator	119,751,932.45
	Number of patients	9640
	Incremental cost per patient	289.78

## Data Availability

All data were extracted from published sources. Formulas extracted from public sources will be available on request, directed to K.S.

## Supplementary Materials

The following supporting information can be downloaded at: https://www.mdpi.com/article/10.3390/jmahp14020033/s1, Figure S1: Estimated sensitivity of intra-cranial bleed detector in India pooling included studies; Figure S2: Estimated specificity of intra-cranial bleed detector in India pooling included studies; Table S1: Unit cost of intra-cranial bleed detector in ambulance; Table S2: Unit cost of intra-cranial bleed detector at CHC; Table S3: Unit cost of intra-cranial bleed detector at tertiary health centre; Table S4: Budget impact analysis at ambulance and CHC level.

## Abbreviations

The following abbreviations are used in this manuscript:

BIA	Budget Impact Analysis
CHC/CHCs	Community Health Centre(s)
CT	Computed Tomography
DHR	Department of Health Research
GBD	Global Burden of Disease
HTA	Health Technology Assessment
HTAIn	Health Technology Assessment in India
ICH	Intracranial Haemorrhage
ICU	Intensive Care Unit
INR	Indian Rupees
LMICs	Low- and Middle-Income Countries
NIRS/NIR	Near-Infrared Spectroscopy
NHM	National Health Mission
RCTs	Randomised Clinical Trials
TBI	Traumatic Brain Injury
