# Supplementary material for: A Decision Tree Cost Analysis of Intracranial Bleed Detection Using a Near-Infrared Device Across Various Healthcare Levels
Source: J Mark Access Health Policy. 2026 Jun 1;14(2):33. doi: 10.3390/jmahp14020033 (PMC13302714; doi:10.3390/jmahp14020033)
Supplement: Supplementary file 1 [file jmahp-14-00033-s001.zip › jmahp-4174493-supplementary.pdf]

Supplementary Figure(s)

Supplementary Figure S1: Estimated sensitivity of intra-cranial bleed detector in India pooling included studies

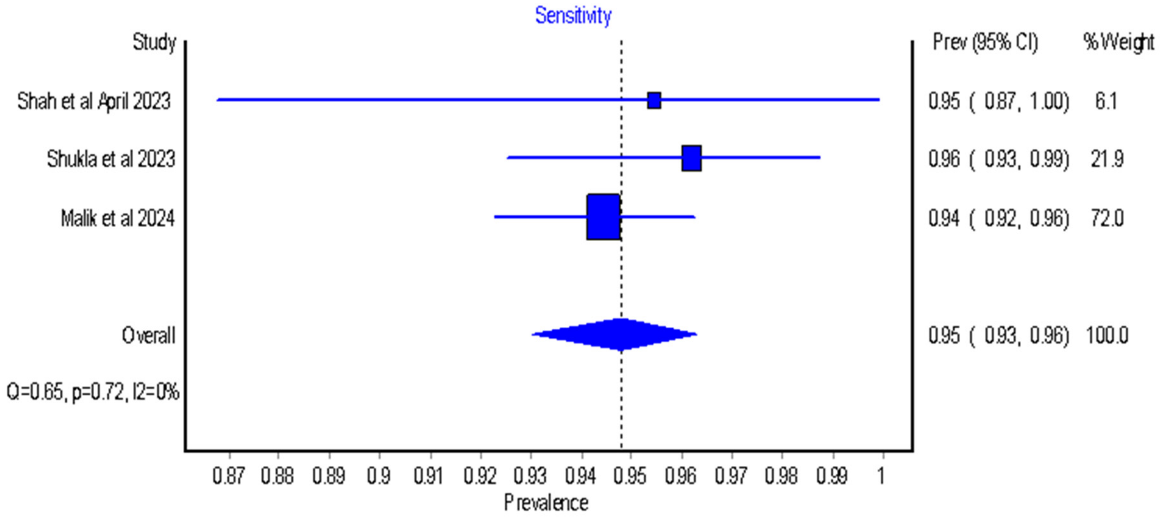

Supplementary Figure S2: Estimated specificity of intra-cranial bleed detector in India pooling included studies

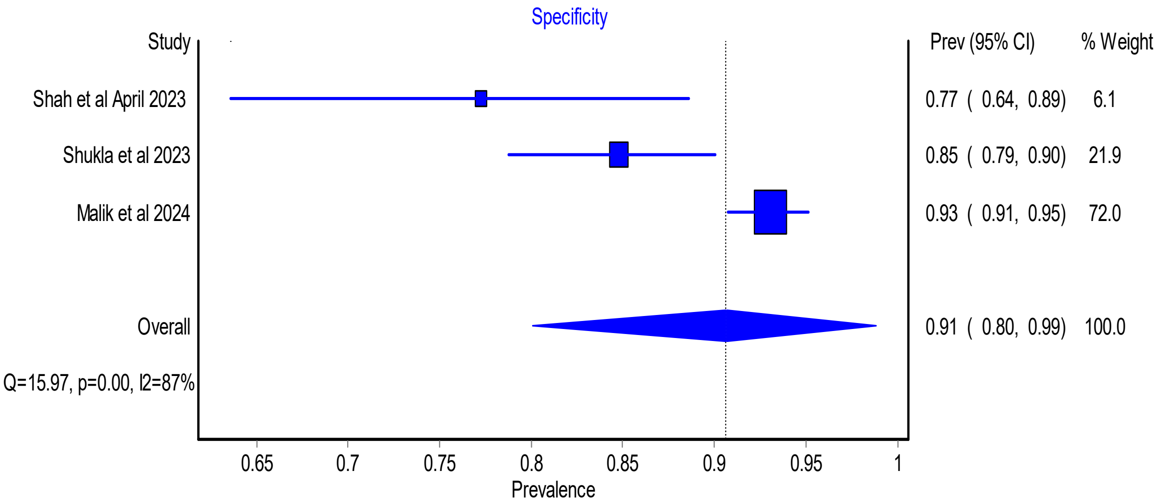

Supplementary Table(s)

Supplementary Table S1: Unit cost of intra-cranial bleed detector in ambulance

Budget head	Quantity	Cost (INR)	Unit cost (INR)	Annual cost (INR)	Reference
Health system cost	1	1,012.47	1,012.47	2,67,292.08	Chauhan et al 2022[21]
Consumables	1	265.30	265.30	70,040.52	From user department
Machine cost	1	13,66,000	13,66,000	1,47,633.79 *	From user department
Training cost Yearly	1	40,000	40,000	40,000.00	From user department
Maintenance yearly	1	50,000	50,000	50,000.00	From user department
Total cost	Rs. 5,74,966.39				
Device cost per mild/moderate TBI cases detected in ambulance	**Rs. 2,177.90**				

* Estimated through annualization

Supplementary Table S2: Unit cost of intra-cranial bleed detector at CHC

Budget head	Quantity	Cost (INR)	Unit cost (INR)	Annual cost (INR)	Reference
Health system cost	1	205.91	205.91	1,76,670.78	Chauhan et al 2022[21]
Consumables	1	265.219	265.219	2,27,557.90	From user department
Machine cost	1	13,66,000	13,66,000	1,47,633.79 *	From user department
Training cost yearly	1	40,000	40,000	40,000.00	From user department
Maintenance yearly	1	50,000	50,000	50,000.00	From user department
Total cost	Rs. 6,41,862.48				
Device cost per mild/ moderate TBI case detected in CHC	**Rs. 748.09**				

* Estimated through annualization

Supplementary Table S3: Unit cost of intra-cranial bleed detector at tertiary health centre

Budget head	Quantity	Cost	Unit cost	Annual cost	Reference
Health system cost	1	338.36	338.36	32,61,790.40	Chauhan et al 2022[21]
Consumables	1	265.133	265.133	25,55,882.12	From user department
Machine cost	1	13,66,000	13,66,000	1,47,633.79*	From user department
Training cost yearly	1	40,000	40,000	40,000.00	From user department
Maintenance yearly	1	50,000	50,000	50,000.00	From user department
Total cost	Rs. 60,55,306.31				
Device cost per mild/ moderate case detected in tertiary centre	**Rs. 628.14**				

* Estimated through annualization

Supplementary Table S4: Budget impact analysis at ambulance and CHC level

**Budget impact at ambulance level**		
1	Population at ambulance level requiring intracranial bleed detection	264
2	Cost of standard of care for intracranial bleed detection at ambulance level	Rs 37,71,011.46
3	New near-infrared device for intracranial bleed detection at ambulance level	Rs 40,30,827.70
4	Incremental cost of near infrared device for intracranial bleed detection at ambulance level	Rs 2,59,816.24
5	Total Number of ambulances in India	17,000
6	**Total Budget Impact Analysis of India at ambulance level (annual cost for India) cost at first year**	**Rs 4,41,68,76,149.28**
7	**Total Budget Impact Analysis of India at ambulance level (annual cost for India) cost at second year**	₹ 4,17,39,47,961.07
8	**Total Budget Impact Analysis of India at ambulance level (annual cost for India) cost at third year**	₹ 3,94,43,80,823.21
**Budget impact at CHC level**		
1	Population at CHC requiring intracranial bleed detection	858
2	Cost of standard of care for intracranial bleed detection in CHC	Rs 1,15,63,758.43
3	New near-infrared device for intracranial bleed detection in CHC	Rs 1,18,73,411.72
4	Incremental cost of near infrared device for intracranial bleed detection in CHC	Rs 3,09,653.29
5	Total number of CHC's in India	6,064
**6**	**Total Budget Impact Analysis of India at CHC level (annual cost for India) cost at first year**	**Rs 1,87,77,37,556.97**
**7**	**Total Budget Impact Analysis of India at CHC level (annual cost for India) cost at second year**	₹ 1,77,44,61,991.34
**8**	**Total Budget Impact Analysis of India at CHC level (annual cost for India) cost at third year**	₹ 1,67,68,66,581.82
